# Man as a Chemical Unit

**Published:** 1911-07

**Authors:** John C. Warbrick

**Affiliations:** Chicago, Ill.


For Texas Medical Journal.

### Man as a Chemical Unit.

BY JOHN C. WARBRICK, M. D., CHICAGO, ILL.

The human body is, practically speaking, a chemical machine, for every part of it is kept in working order, one can almost say, by the aid of chemical means, while progressive changes are not only going on all of the time in the ages of different individuals from youth to old age, thus affecting the body parts, but chemical changes are also continually going on within the body, affecting the organs from youth to old age. The internal organs of the body each performing its separate function, keep all of the vital processes in a state of normal or healthy activity.

Thus man "lives and has his being" by the breathing in of the oxygen of the air to sustain life wherever he goes and the giving out of carbonic acid gas in return preserves the chemical unity of the body at all times, and by this means man proceeds on his way through life. As he is moved wherever he wants to go by his muscular system acting under the control of his will power, subject to reason and judgment, so wherever he roams he maintains his good health by the breathing in of the oxygen of the air, and is able to accomplish whatever he sets out to do, provided nothing unforeseen overtakes him.

In winter, in order to maintain the warmth of the body internally and escape the cold, man clothes himself with heavier raiment, while in summer, in order to aid the evaporation from the pores of the skin and to keep the body cool, light clothing is worn, and thus the body is kept in a state of chemical equilibrium by means of these external agents, regulated by atmospheric temperatures. When any organic changes occur, as in heart disease, the chemical equilibrium of the body is disturbed and compensation must be given while aeration of the blood is more difficult and man can not range at leisure in all atmospheric densities as in the normal state, without at once being affected in some degree.

It is the same in tuberculosis, where even greater compensation must take place, as the organs concerned in the vital act of breathing being attacked, aeration of the blood is more difficult and the various atmospheric densities produce a greater effect upon the body.

Man's range has a wide limit, extending, as it mostly does, over the surface of the earth, but also under the water, through the air and under the ground, being limited by no country, nor climate, nor law in the normal state. Thus he lives and thrives in various media, and under variable conditions, while the chemical equilibrium of the body adapts itself to the media in which it circulates. As necessity demands, man roams for sustenance and propagates his specie by marriage, as he desires. He cultivates the ground, grows wheat, vegetables and other products of the soil, for his need under various climatic conditions.

The densities of the air and temperature with which man comes in contact, are regulated by the lungs, almost like a thermometer in the interchange of gases which occurs there when the blood is re-oxygenated, and the vitality of the tissue renewed while foul gases are given off in return.

The climatic conditions of the globe being due to the thermal influences of the ocean currents, man can roam as he will, his body adapting itself to all climates and countries alike wherever he elects to travel over the earth.

While the vital processes of the body then are regulated by the physical attributes of nature, such as light, heat, cold, air, water and climate, human beings are affected differently by these ever-varying physical conditions. As man is of the highest order of intelligence he is different from all other animals which inhabit the universe in his habits, mode of living and environment.

The birds of the air, whose blood is warmer than that of man's, enables them to soar to great heights in the sky, while their covering of feathers serves as a good non-conductor of heat, maintaining the internal warmth of the body in the atmosphere, and thus the birds, as created by nature, are well adapted to all aerial conditions, but as governed by instinct whenever necessary, they migrate from one climate to another to escape the cold, being able to undertake long journeys through the air without rest or food, and often can be seen in large numbers sweeping through the air at some height outlined against the sky in the form of long chains or columns.

Man also tends to imitate the birds of the air by the conquest of aerial navigation and the adapting of himself to aerial conditions by soaring to high altitudes for a long time, and journeying to considerable distances by balloon or other suitable craft, and thus another field has been conquered and added to the victories already won by him.

*Finale:* Finally all over the world, the gigantic processes and marvels of nature gently unfold their hidden secrets to the subtle intellect, the continuous energy, the intrepid daring of man by which products, and under whose climatic conditions he manages to exist.

